# The processed *Sanguisorba officinalis* L. triterpenoids prevent colon cancer through the TNF-*α*/NF-κB signaling pathway, combined with network pharmacology, molecular simulation dynamics and experimental verification

**DOI:** 10.3389/fimmu.2025.1605326

**Published:** 2025-06-18

**Authors:** Chunli Gan, Yuanqiu Mu, Shah Syed Faizan Ali, Xuepeng Shi, Shuang Jiang, Zhengyang Wang, Xiaotian Wu, Xiaotong Wang, Zhibin Wang, Shulu Zhang, Zheng Feng, Chunjuan Yang

**Affiliations:** ^1^ Department of Medicinal Chemistry and Natural Medicine Chemistry, College of Pharmacy, Harbin Medical University, Harbin, China; ^2^ Department of Pharmaceutical Analysis and Analytical Chemistry, College of Pharmacy, Harbin Medical University, Harbin, China; ^3^ Key Laboratory of Chinese Materia Medica (Ministry of Education), Heilongjiang University of Chinese, Harbin, China

**Keywords:** *Sanguisorba officinalis l.*, processed, colon cancer, TNF-α/NF-κB, COX-2, iNOS

## Abstract

**Background:**

*Sanguisorba officinalis* L. (S.L.), a traditional Chinese medicine from the Rosaceae family, is recognized for its rich content of triterpenoids, which are known for their antioxidant, anti-inflammatory, and anti-tumor properties. Although its traditional uses and biological activities are well known, its role in preventing colon cancer and the underlying mechanisms remain unclear. This study aims to elucidate the preventive mechanisms of triterpenoids in both raw (TR) and processed (TP) forms of S.L. against colon cancer.

**Methods:**

The AOM/DSS-induced mouse model of colon cancer was employed to elucidate the mechanism underlying the preventive effects of *Sanguisorba officinalis* L. triterpenoids (ST) against colon cancer. A comprehensive suite of techniques, including hematoxylin-eosin staining (H&E), immunohistochemistry (IHC), TUNEL staining, Western blotting (WB), and DNA methylation analysis, was utilized to investigate the preventive effects of ST on colon cancer. The main active compounds were identified using UPLC-Q-TOF-MS, and potential active compounds were screened through network pharmacology and molecular docking. The stability of the protein-ligand complexes was further assessed using molecular dynamics simulations.

**Results:**

*In vivo* experiments, treatment with ST significantly improved the clinical manifestations, Disease Activity Index (DAI) scores, and pathological lesions associated with colon cancer, with all drug administration groups outperforming the model group. Additionally, ST markedly enhanced gut barrier function by downregulating the levels of TNF-*α*, p65, COX-2, and iNOS. Furthermore, ST dramatically ameliorated the colonic immune-inflammatory state, which was associated with decreased expression of proliferative proteins and increased expression of apoptotic proteins. Among the identified triterpenoids, compound 27 May be the main active compound. Notably, compound 27 can form a stable complex with TNF-*α*.

**Conclusion:**

These results suggest that TP has a more pronounced colon cancer prevention effect than TR. TP play a role in preventing colon cancer by down-regulating TNF-*α* and thereby inhibiting the NF-κB signaling pathway. This research not only fills the mechanism gap of S.L. in the field of colon cancer prevention, but also provides methodological support and theoretical foundation for its transition from traditional Chinese medicine to clinical practice through the integration of multi-disciplinary technologies and the verification of precise targets.

## 1 Introduction

*Sanguisorba officinalis* L. (S.L.), is a traditional Chinese medicine and a member of the Rosaceae family. There are over thirty varieties of elm worldwide and they are widely distributed, mainly concentrated in Western Europe and the northern temperate zone of Asia. There are seven genuine products in China. They are respectively *Sanguisorba officinalis* L., *S. tenuifolia Fisch*., *S. filiforms (*Hook. f.*) Hand. Mazz*., *S. Alpine Bge*., *S. applanata* Yu et Li., *S. Diandra Wall. ex Hoedb*. and *S. Sitchensis Mey.* And six variants, they are respectively *S. officinalis* L. var. *glandulosa* (Kom.) Worosch., *S. officinalis* L. var. *carnea* (Fisch.) Regel. ex Maxim., *S. officinalis* L. var. *longifolia* (Bert.) Yü et Li, *S. officinalis* L. var. *longiflila* (Kitagawa.) Yu et Li, *S. tenuifolia* Fisch. var. *alba* Trautv. et Mey. and *S. applanata* var. *Applanate* Yü et Li. According to the Chinese pharmacopeia, it plays a major role in the treatment of hematochezia, bleeding hemorrhoids, bloody flux, metrorrhagia and metrostaxis, bleeding wounds, burns and scalds, and swollen carbuncles. Besides, *in vivo* and *in vitro* studies have illustrated that plants from the *Sanguisorba officinalis* (*S. officinalis*) present a wide range of pharmacological properties, including anti-tumor and immunomodulating activities (1). Simultaneously, it has been reported that *S. officinalis* has an obvious anti-tumor effect, which inhibits the growth of human colon cancer cells HT-29, HCT116, RKO, SW480, and SW620 cells (2–4). The main chemical constituents isolated from *S*. off*icinalis* include triterpenoids, tannins, flavonoids, etc. Triterpenoids are the main components of *S. officinalis*, the pharmacological studies mainly focus on antioxidant, anti-inflammatory, and anti-tumor activities in nearly a decade (5).

The World Health Organization’s 2024 Epidemiological Report shows that both the incidence and mortality of colorectum cancer are increasing year by year. The risk of this type of cancer has been associated with complex interactions among inherited susceptibility, increasing age, and environmental factors, including lifestyle factors such as diet and physical activity. Long-standing UC and Crohn’s colitis (except limited proctitis) increased patients’ risk of colon cancer by 2–3 times (6). Although there are advances in surgery, radiation therapy, and chemotherapy to treat colorectal cancer, new treatment alternatives related to chemoprevention are necessary. Several studies have focused on evaluating the control of carcinogenesis and identifying naturally occurring dietary agents with the capacity for inhibiting, retarding, or reversing the preneoplastic lesions, rather than curing the end-stage disease (7). *In vitro* and *in vivo* studies have reported that dietary bioactive compounds have preventive effects against the pathogenesis of colon cancer by suppressing inflammation and cell proliferation (8). Natural products have been shown to inhibit the NF-κB signaling pathway by down-regulating TNF-*α* (9). In addition, NF-κB plays a critical role in regulating the immune response, deregulated NF-κB activation could facilitate the pathogenic processes of inflammatory diseases such as colitis-associated colorectal cancer through transcription of mainly proinflammatory and anti-apoptotic target genes (10). The use of azoxymethane (AOM) and dextran sodium sulfate (DSS) in mice has become an exceptional model for the study of colon carcinogenesis *in vivo* due to its high reproducibility, as well as its affordable and straightforward administration (11). S.L. has received considerable attention for modulating inflammation and protecting against cancer development (3, 12).

**Figure 1:**
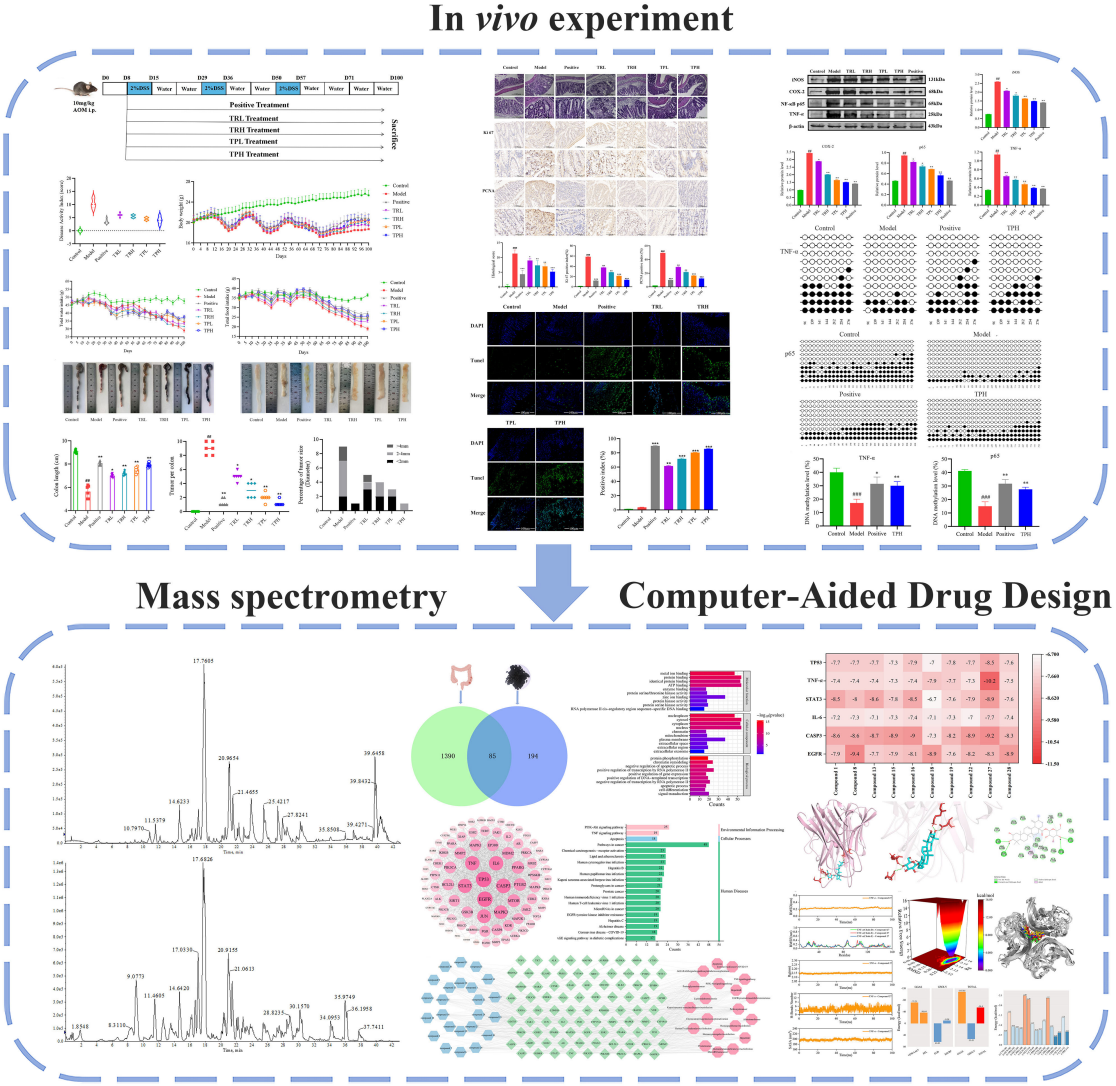
The flowchart of this study. (Model vs Control: ^##^ *P*<0.01, ^###^ *P*<0.001; Positive, TRL, TRH, TPL, TPH vs Model: *P<0.05, **P<0.01, ***P<0.001).

Epidemiological studies have suggested that the intake of rosaceae natural medicine decreases the risk of developing colorectal cancer (13). Among Rosaceae, *S*. *officinalis* has been recognized as a natural medicine with health benefits (14). However, the chemopreventive effect of *S. officinalis* on the gene expression and regulation of inflammatory markers in colon cancer *in vivo* has not yet been explored. The objective of this study was to evaluate the effects of different doses of TR and TP in colon cancer models, in addition to identifying main active compounds. The flowchart of this study is shown in **Figure 1**.

Current studies have shown that the water extract of S.L. can activate the reactive oxygen-mediated mitochondrial-Caspase-dependent apoptotic pathway and trigger apoptosis (15). In addition, S.L. can also induce cell death through autophagy and inhibit the proliferation of colon cancer cells through G_0_-G_1_ phase cell cycle arrest mediated by the Wnt signaling pathway (16). *In vivo* experiments, the water extract of S.L. also demonstrated a good anti-tumor effect and was able to inhibit the migration and invasion of colon cancer cells by suppressing the Wnt/*β*-catenin signaling pathway (17). In our previous research, it has been found that processed S.L. can induce apoptosis in colon cancer cells (4). This study systematically revealed for the first time that ST and its processed products exert chemopprophytic effects on colon cancer by inhibiting the TNF-*α*/NF-κB signaling pathway. It not only clarified the key chemical components by which processing enhances the efficacy of drugs, but also established a direct connection between traditional applications and modern mechanism research, filling the research gap in this field.

## 2 Materials and methods

### 2.1 Materials and reagents

S.L. was purchased from Harbin, Heilongjiang Province. DSS (MW: 36,000-50,000) and AOM was acquired from MP Biomedicals (Santa Ana, CA, USA). RIPA, Pmsf, and Removal solution of Western primary and secondary antibodies were acquired from Thermo Fisher Scientific. Formalin was purchased by Sigma-Aldrich. Hematoxylin and eosin were purchased from Hangzhou Hua ‘an Biotechnology Co., LTD. 4’, 6-diamidino-2-phenylindole was purchased from Meilen Bio. TNF-*α*, COX-2, NF-κB p65, and iNOS were obtained from ABclonal. The specific antibodies applied for immunohistochemistry were from Affinity Biosciences (Cincinnati, OH, USA).

### 2.2 Samples and processing

S.L. processed according to the Chinese Pharmacopoeia 2020 edition. The processed and raw are crushed with a high speed grinder, then passed through an 80–100 mesh sieve, sealed and stored.

### 2.3 Extraction technique and enrichment of TR and TP

The raw and processed S.L. were extracted by heating reflux extraction method (1 hour, 3 times), the extraction solution was 70% ethanol solution, the liquid-solid ratio was 8:1, and the extract was concentrated into extractum under vacuum reduction. According to previous reports (18), the contents of TR and TP were determined by perchloric acid colorimetry. The standard curve is plotted as follows: *y*=0.06921*x*-0.0059 (*R*=0.9995) (where *x* is the mass concentration (mg/mL) value and *y* is the absorbance value). TP and TR are dissolved by water. Dissolved TR and TP were extracted three times with n-hexane and then three times with saturated n-butanol. The obtained n-butanol layer was concentrated to obtain enriched TR and TP. Triterpenoids in TR and TP were 62.5% and 73.9%, respectively.

### 2.4 Animals administration of TR and TP

**Figure 2:**
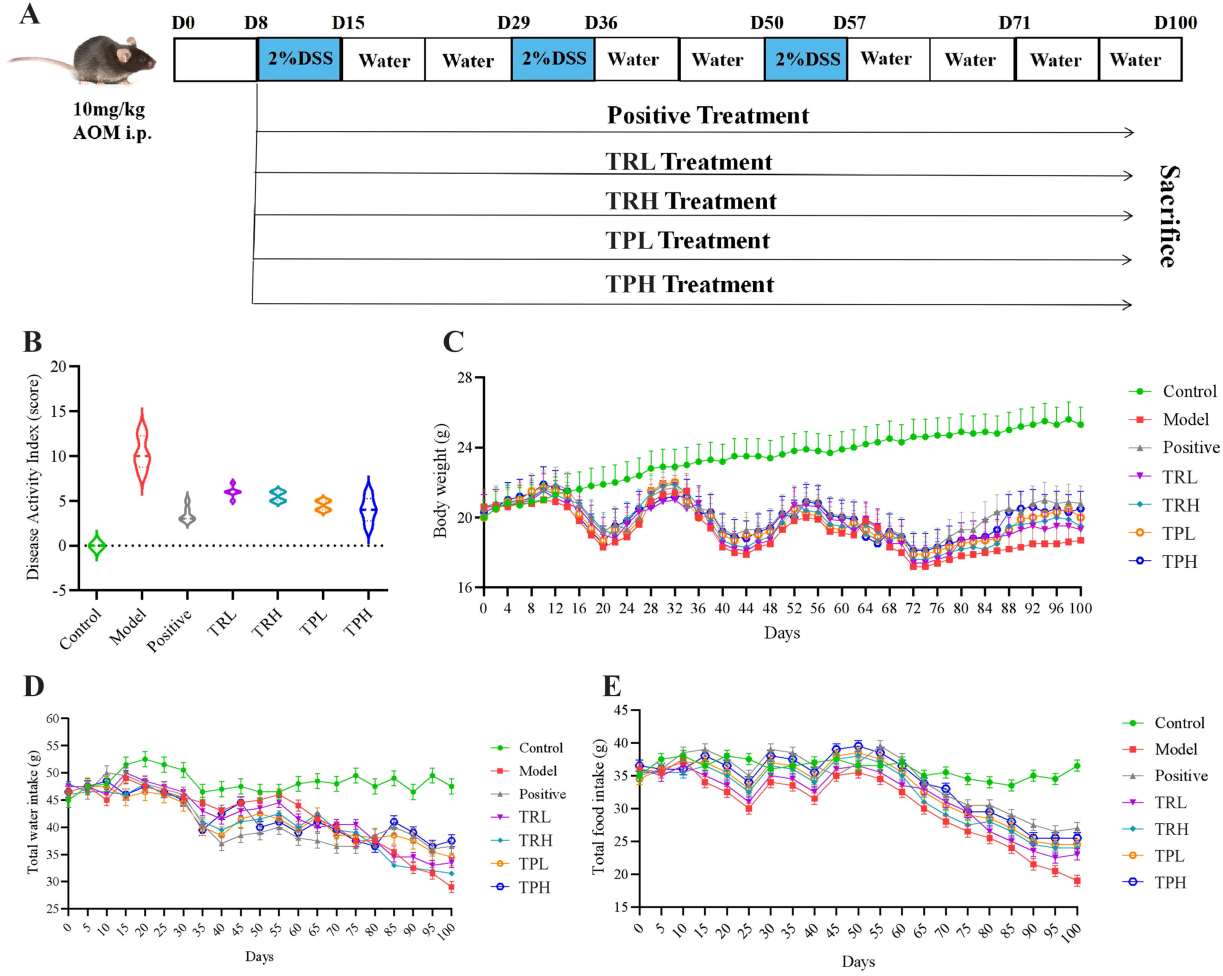
General status of mice in each group. **(A)** AOM/DSS induced colon cancer model. **(B)** Disease Activity Index, DAI score. **(C)** The trend of weight change in each group of mice. **(D)** The trend of water intake in each group of mice. **(E)** Trends of food intake in each group of mice.

C57BL/6 male mice (*n*=84, 6 weeks old) were obtained from “Liaoning Changsheng Biotechnology Co., LTD.”. After a 1-week period of acclimatization (12-h dark/light cycle at 25 °C), mice were randomly distributed into seven groups (*n*=12, each mouse used an individual cage system). Qualification certificate number: SCXK (Liao) 2023-0001. The model building scenario is shown in **Figure 2A**. Based on conversion and extraction rate calculation, the doses of TR and TP are as follows: the low dose is 15mg/kg and the high dose is 30mg/kg. All the mice were anesthetized and sacrificed after anesthetizing.

### 2.5 Disease activity index evaluation

The DAI score is calculated according to the previous criteria (19).

### 2.6 Histopathology analysis

Hematoxylin and eosin (H&E) staining sections were performed according to previous reports (20). Immunohistochemical staining of sections was performed according to manufacturer’s instructions. Apoptosis detection kits were used for TUNEL staining.

### 2.7 Western blot analysis

Experiments were conducted according to literature reports (20). Image J software is used for quantitative analysis.

### 2.8 DNA methylation analysis

Bisulfite sequencing PCR was used for DNA extraction and detection of colon cancer-related genes. Firstly, 1 μg of genomic DNA was converted to bisulfite, purified, and recovered, and relevant primers were designed. Subsequently, we purified mouse colon tissue using the DNA extraction kit, bisulfite treatment kit, etc. The purified products were amplified by PCR, and all the amplified PCR products were loaded into the agarose gel wells for electrophoresis. Generay’s pTG19-T was used as the vector, following the product instructions in the kit, and positive plasmids were sequenced. The sequencing result analysis software used in this study includes the sequencing instrument ABI3730XL, sequencing reagent BigDye V3.1, and sequencing analysis software Sequence Analysis V5.02.

### 2.9 UPLC-Q-TOF-MS analysis of the chemical composition of TP

The accurate TP weighing of 2.1 mg obtained in 2.3 was contained in a 10 ml volumetric bottle. The TP solution was filtered through 0.22 μm membrane and stored in refrigerator at 4°C. The chromatography was performed on Waters ACQUITY UPLC BEH C^18^ column (2.1 mm×100 mm, 1.7 μm, American Waters Company). The liquid phase conditions were column temperature 40°C, sample chamber temperature 4°C, sample 3 μl, flow rate 0.3 mL·min^-1^. The mobile phases were distilled water with 0.1% formic acid (A) and acetonitrile (B). The program was applied to gradient elution: 0–2 min, 95%-95% (A); 2–25 min, 95%-50% (A); 25–42 min, 50%-5% (A). Electrospray ion sources (ESI) are used. High-purity N_2_ is used as atomizing gas and cone-hole gas. The mass spectrum parameters were as follows: atomized gas pressure, 650 kPa; scanning range, 50~1500 Da; temperature, 550°C; positive ion spary voltage, 5500 V; negative ion spary voltage, -4500 V; curtain gas, 35 psi; ion source gas 1, 50 psi; and ion source gas 2, 50 psi; declustering potential, ± 80eV; collision energy, ± 35 eV.

### 2.10 Network pharmacology analysis

Drug targets from SwissTargetPrediction (https://www.swisstargetprediction.ch/) in the database access (probability>0). colon cancer targets were collected from GeneCards (https://www.genecards.org/) and OMIM databases (https://www.omim.org/). Subsequently, potential active ingredient targets and colon cancer targets were imported into the bioinformatics to make Venn diagram (https://bioinformatics.psb.ugent.be/webtools/Venn/) to collect common targets. The common targets were then imported into STRING database (https://cn.string-db.org) to obtain protein-protein interaction (PPI) and visualize by Cytoscape 3.10.2. DAVID (https://david.ncifcrf.gov/home.jsp) was employed to perform Gene ontology (GO) enrichment and Kyoto Encyclopedia of genes and Genomes (KEGG) pathway analysis. Finally, “component-target-pathway” network of network pharmacology was constructed using Cytoscape 3.10.2.

### 2.11 Molecular docking

The crystal structure of the core targets were obtained from the protein database (pdb) (https://www.rcsb.org/). The small molecule structure is converted into pdb format by Chem3D software. The ligands will be dehydrated and hydrotreated. The ligands and receptors were converted to pdbqt format using Autodock 1.5.7. Molecular docking Performed by Autodock Vina to obtain the Active ingredients and the key targets. Finally, PyMOL 4.6.0 and Discovery Apply Studio 4.5 to visualize the results.

### 2.12 Molecular dynamics simulation

Compound 27 and TNF-*α* were simulated by molecular dynamics according to the previously reported method (21). Finally, we analyzed the root mean square deviation (RMSD), root mean square fluctuation (RMSF), radius of gyration (Rg), the number of hydrogen bonds between protein and ligand, relative free energy distribution and 0,25,50,75,100 ns in the molecular dynamics simulation trajectory of the TNF-*α* - compound 27 complex. In addition, we used the MM/GBSA method to calculate the average binding free energy between protein and ligand.

### 2.13 Data analysis

Data were presented as mean ± standard deviation (SD). Student’s t-test and one-way ANOVA were used for comparisons between and among different groups, respectively. *P* ≤ 0.05 was considered statistically significant. Histograms were plotted using Graph Pad Prism (version 6.0; GraphPad Software Inc., CA, USA).

## 3 Results

### 3.1 Evaluation of the preventive effects of TR and TP on colon cancer in mice

To investigate the potential therapeutic effects of TR and TP on colon cancer, a model was established by administering 2% DSS over three cycles to induce colon cancer. The symptoms associated with chronic colitis, including weight loss, diarrhea, and hematochezia, were recorded for 100 days to calculate the DAI. ST treatment significantly reduced the DAI compared to the untreated model group (**Figure 2B**), indicating a mitigation of colitis severity. Furthermore, ST administration attenuated body weight loss and even promoted weight recovery in treated mice, suggesting a protective role against colitis-associated colon cancer progression (**Figure 2C**). Additionally, as illustrated in **Figures 2D, E**, the control group maintained stable food and water intake throughout the study. In contrast, compared with the treatment group, the food consumption and water intake of the model group were significantly reduced. These findings further support the conclusion that ST ameliorates DSS-induced colon cancer by improving metabolic and clinical parameters.

### 3.2 Effect of TR and TP on colon length and tumorcondition in mice

**Figure 3:**
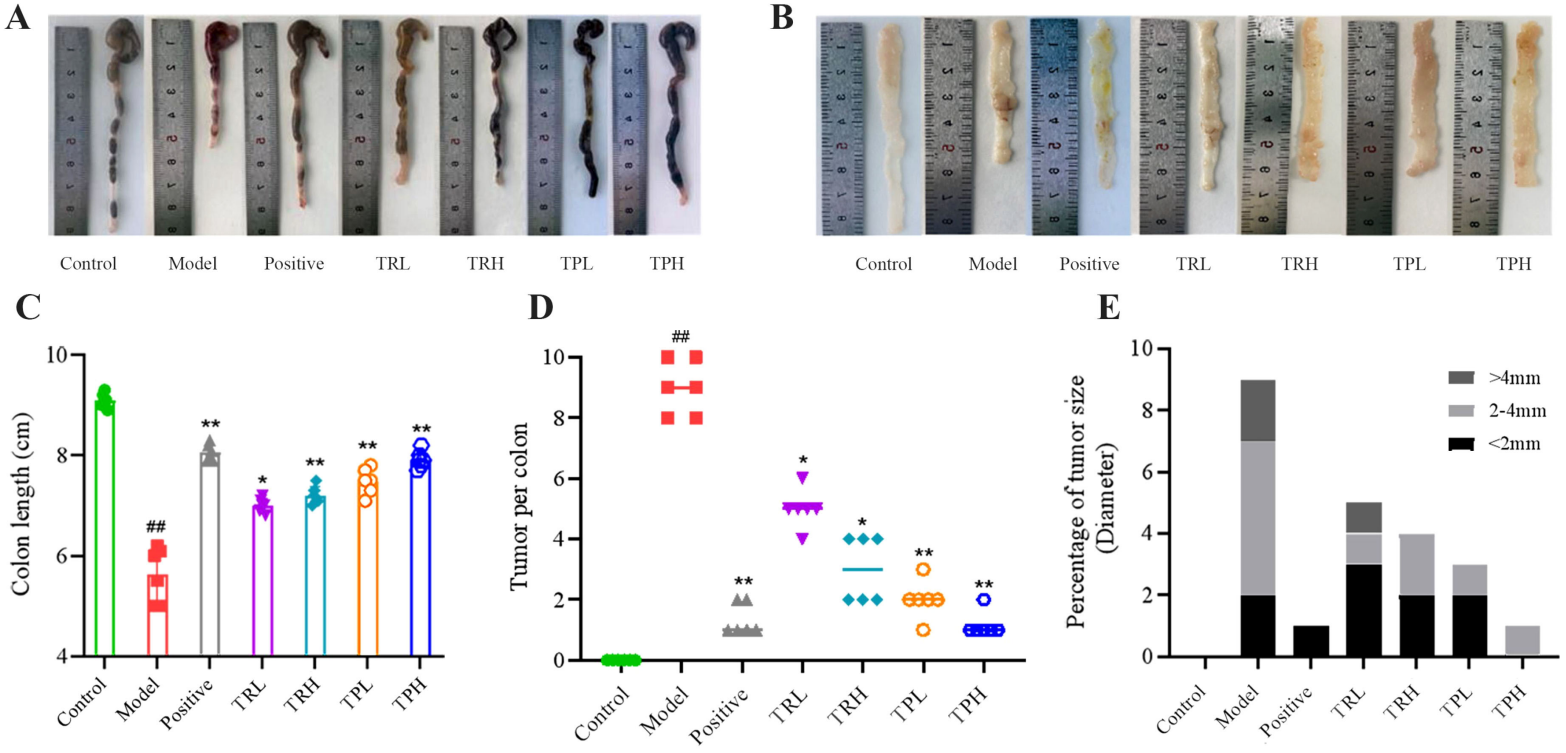
Observation and statistical results of colonic tissue appearance in each group of mice. **(A)** Colon length appearance. **(B)** Appearance of colon tissue. **(C)** Colon length statistics. **(D)** Tumor number statistics. **(E)** Tumor size statistics. (Model vs Control: ^##^ *P*<0.01; Positive, TRL, TRH, TPL, TPH vs Model: *P<0.05, **P<0.01; *n*=6).

At the termination of the experiment, mice were euthanized, and colon tissues were collected for macroscopic and morphometric analysis. The control group exhibited normal colonic morphology, characterized by a smooth, intact surface and typical coloration. In contrast, the model group displayed pronounced pathological alterations, including reddish-brown discoloration, disorganized architecture, severe congestion, and significant colon shortening (**Figure 3A**). Quantitative assessment revealed that the mean colon length in the control group was 9.10 ± 0.15 cm, whereas the model group demonstrated a marked reduction (5.50 ± 0.56 cm, *P*<0.01). Treatment with TR and TP significantly attenuated this shortening (*P*<0.05), indicating a protective effect against DSS-induced structural damage (**Figure 3C**). Tumor incidence and morphology were further evaluated (**Figure 3B**). The number of tumors in the model group was 9.00 ± 0.89, the number of tumors in the positive group was 1.16 ± 0.51, the number of tumors in the TRL group was 5.00 ± 0.63, the number of tumors in the TRH group was 3.00 ± 1.09, and the number of tumors in the TPL group was 2.00 ± 0.63. The number of tumors in the TPH group was 1.33 ± 0.40, significantly different from that in the model group (*P*<0.05), and the statistical results are shown in **Figure 3D**. Notably, tumors in treated mice were predominantly solitary and smaller in diameter (<2 mm), with a minority measuring 2–4 mm and rare instances exceeding 4 mm (**Figure 3E**). These findings suggest that both TR and TP exert dose-dependent chemopreventive effects against colon cancer, albeit with varying efficacy.

### 3.3 Histopathological effects of TR and TP on mice with colon cancer

**Figure 4:**
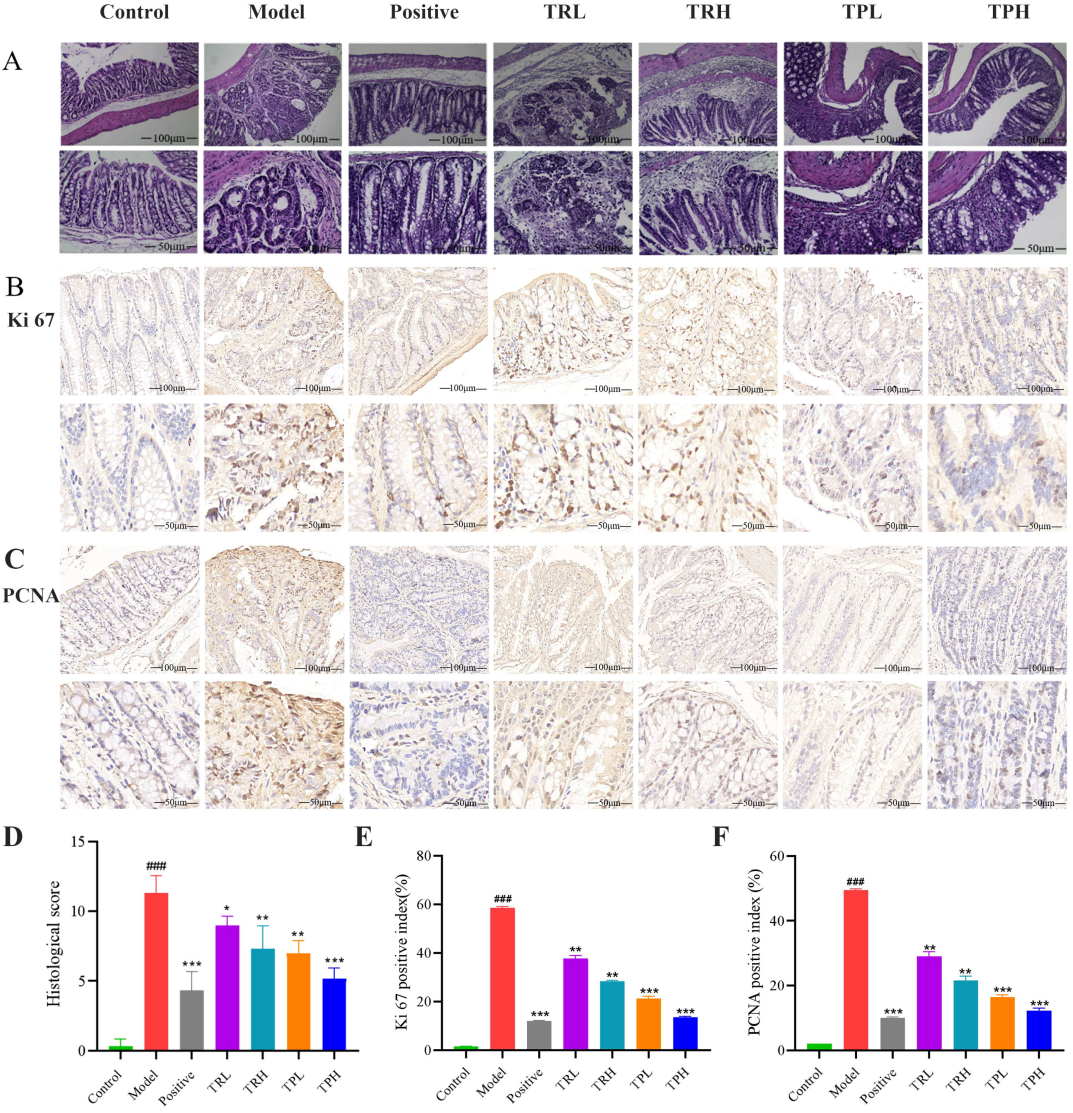
HE staining results of colon tissue of mice and the detection of colon cancer proliferation index. **(A)** The results of HE staining pathology. **(B)** The results of immunohistochemistry (IHC) stains for Ki67. **(C)** The results of immunohistochemistry (IHC) stains for PCNA. **(D)** Histological score. **(E)** Ki67 labeling index of mice in each group. **(F)** PCNA labeling index of mice in each group. (Model *vs* Control: ^###^ *P*<0.001; Positive, TRL, TRH, TPL, TPH *vs* Model: ^*^ *P*<0.05, ^**^ *P*<0.01, ^***^ *P*<0.001; *n*=3).

In the model group, the glandular structures exhibited back-to-back and cribriform patterns, with severe inflammatory infiltration, crypt loss, and near-total depletion of the epithelial mucus layer. Goblet cells were absent, nuclei were enlarged and hyperchromatic, the nucleus-to-cytoplasm ratio was increased, nuclear polarity was lost, and multinucleation occurred. Mucosal folds were disrupted, and the colon contour became irregular. TR and TP can protect the integrity of the colonic mucosal structure. Due to the differences in the dosage and composition of the drug, the degree of damage to the colonic mucosa varies slightly, and the results are shown in **Figure 4A**. Histological scoring revealed significant differences between the model group and the control group (*P*<0.005), as well as between the model group and the drug administration groups (*P*<0.05). The statistical results are presented in **Figure 4D**.

**Figure 5:**
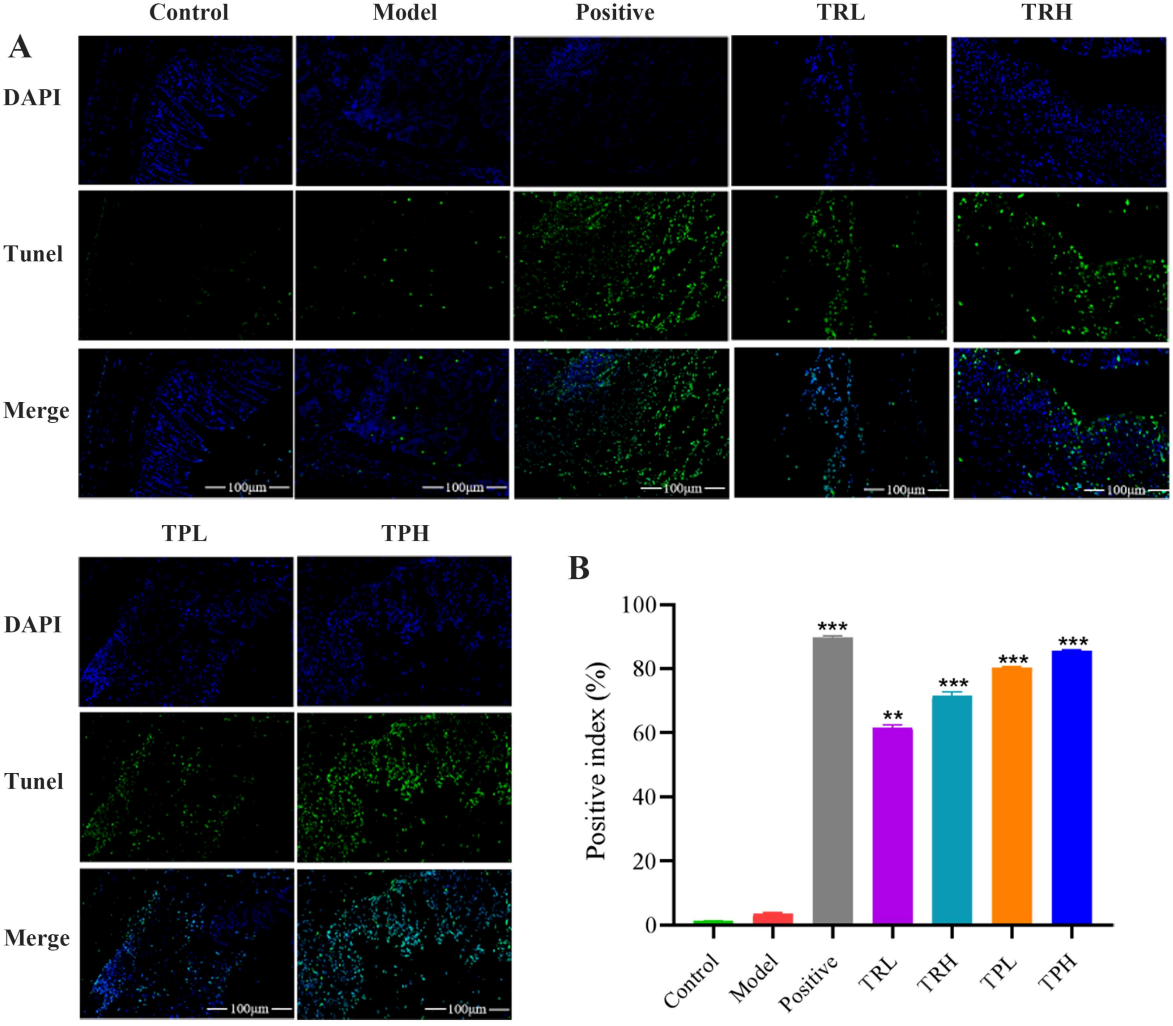
Expression of TUNEL immunofluorescent in tumor tissue of colon cancer mice. **(A)**TUNEL histochemical images of colon cancer mice. **(B)** Results of semi-quantitative analysis. (Scale bar=100 μm. Positive, TRL, TRH, TPL, TPH vs Model: **P<0.01, ***P<0.001; *n*=3).

In the model group, the expression of Ki67 in colon tissues was significantly elevated, with a positive cell index of 58.50 ± 0.60% (**Figure 4B**). In contrast, the number of Ki67-positive cells decreased in a dose-dependent manner in the TRL, TRH, TPL, TPH, and positive control groups, with positive cell indices of 37.70 ± 1.20%, 28.30 ± 0.40%, 21.20 ± 0.95%, 13.50 ± 0.35%, and 12.02 ± 0.25%, respectively. Semi-quantitative analysis of Ki67 protein expression (**Figure 4E**) revealed that, compared to the model group, Ki67 expression was significantly reduced in the TRL, TRH, TPL, and TPH groups (*P*<0.01). Notably, the efficacy of TPH in reducing Ki67 expression was comparable to that of the positive control group. Similarly, the expression of PCNA was significantly higher in the model group compared to the control group, with a positive cell index of 49.50 ± 0.40% (**Figure 4C**). The expression of PCNA was also reduced in a dose-dependent manner in the TRL, TRH, TPL, TPH, and positive control groups, with positive cell indices of 29.00 ± 1.45%, 21.50 ± 1.30%, 16.40 ± 0.75%, 12.20 ± 0.88%, and 10.01 ± 0.25%, respectively (**Figure 4F**). The reduction in PCNA expression was statistically significant (*P*<0.01). TUNEL staining was used to assess apoptosis in colon tissues. As shown in **Figures 5A, B**, the apoptotic fluorescence intensity was significantly higher in the TRL, TRH, TPL, TPH, and positive control groups compared to the model group (3.50 ± 0.40%). The fluorescence intensities in these groups were 61.60 ± 0.80%, 71.50 ± 1.30%, 80.40 ± 0.26%, 85.50 ± 0.35%, and 89.90 ± 0.40%, respectively (*P*<0.01). In the control and model groups, few green fluorescent cells were observed, indicating minimal apoptosis. In contrast, a significant increase in apoptotic cells was observed in the colon tissues of mice treated with TR and TP. These findings demonstrated that both TR and TP could inhibit the proliferation of colon tumor cells in a dose-dependent manner, with TP exhibiting a more pronounced effect than TR.

### 3.4 Effects of TR and TP on the expression of key proteins in NF-κB signaling pathway

**Figure 6:**
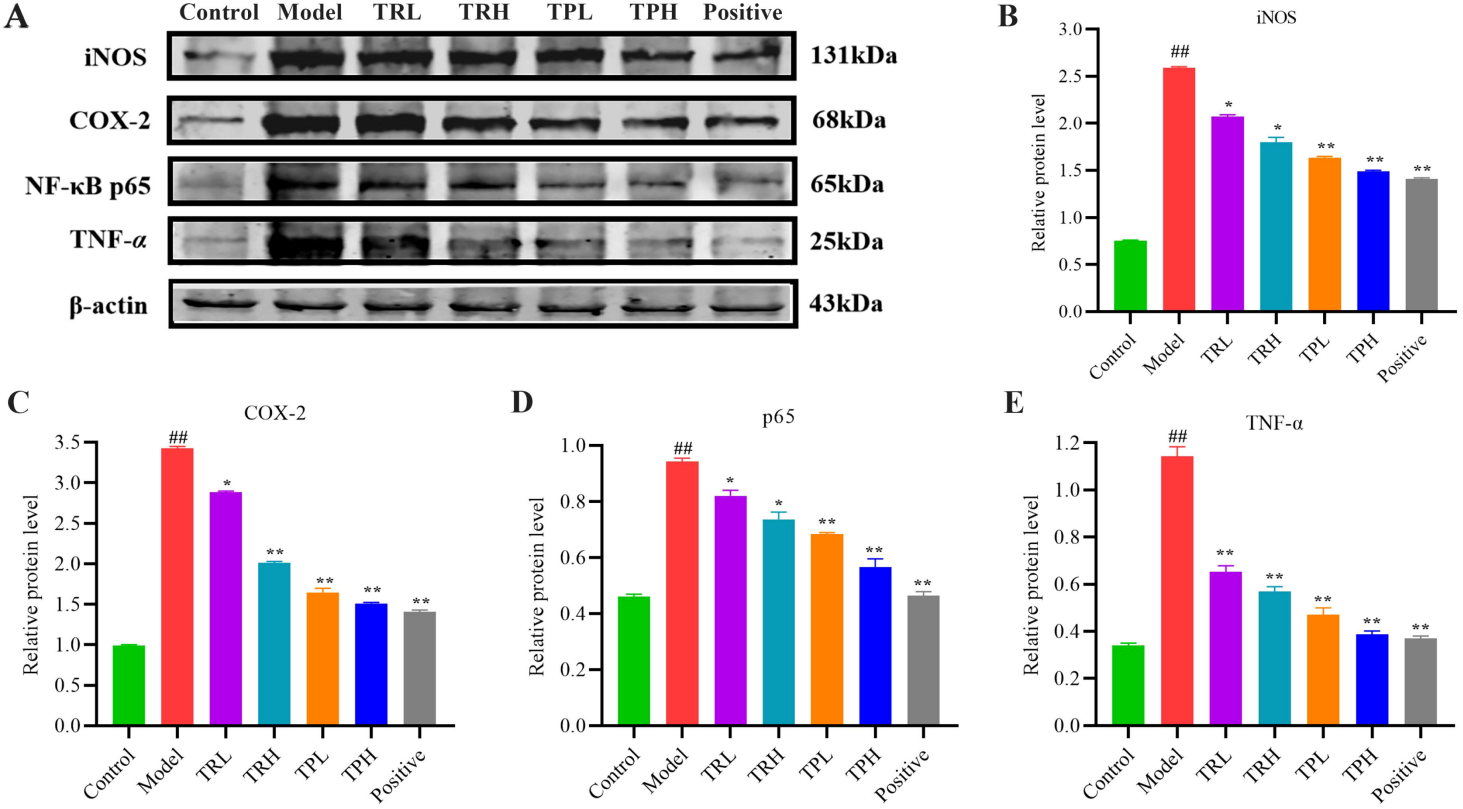
Effect of TR and TP on expression of key proteins in NF-κB signaling pathway. **(A)** Western-blot results. **(B)** Results of iNOS statistical analysis. **(C)** Results of COX-2 statistical analysis. **(D)** Results of p65 statistical analysis. **(E)** Results of TNF-*α* statistical analysis. (Model vs Control: ^##^ *P*<0.01; Positive, TRL, TRH, TPL, TPH vs Model: *P<0.05, **P<0.01; *n*=3).

The underlying inhibitory mechanisms of TR and TP on colon cancer cells were further elucidated through Western blot analysis (**Figure 6**). Compared with the control group, the model group exhibited a significant upregulation in the protein levels of TNF-*α*, NF-κB p65, COX-2, and iNOS (*P*<0.005). Upon treatment with TRL, TRH, TPL, TPH, and the positive control, a dose-dependent decrease in the expression of these proteins was observed. Specifically, significant reductions were noted when comparing the treatment groups with the model group (*P*<0.05). These findings indicate that the triterpenoid components of both TR and TP effectively inhibited the expression of TNF-*α*, NF-κB p65, COX-2, and iNOS in a dose-dependent manner. Furthermore, TP demonstrated a more significant inhibitory effect compared to TR, which might imply that certain specific triterpenoids compound components in TP have stronger biological activity, or that the combination of triterpenoids compound components in TP has a synergistic effect in inhibiting inflammatory signaling pathways.

### 3.5 DNA methylation analysis of TNF-*α* and NF-κB p65 genes

**Figure 7:**
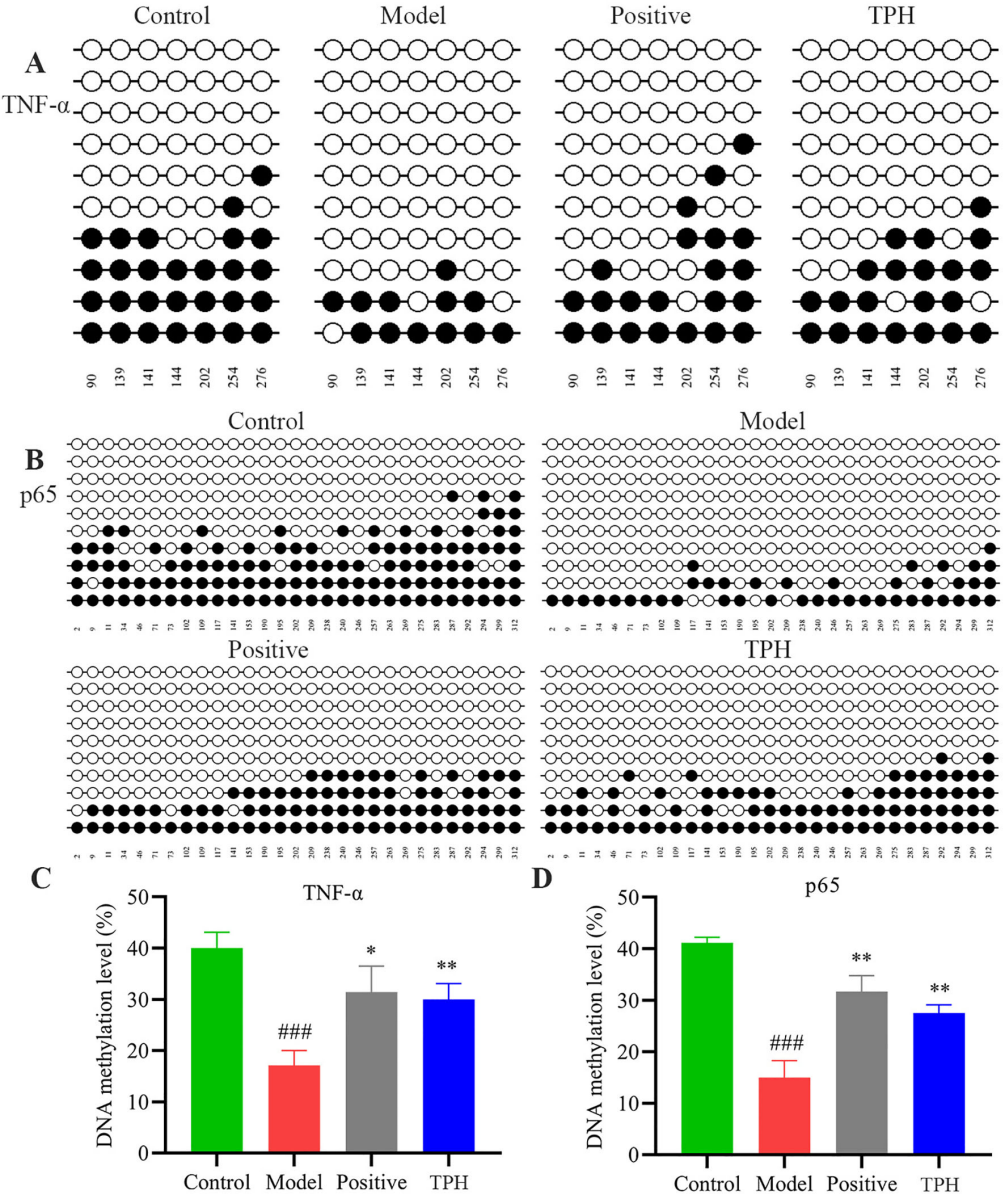
The results of TNF-α and p65 DNA methylation. **(A)** TNF-α DNA methylation results. **(B)** p65 DNA methylation results. **(C)** Results of TNF-α DNA methylation statistical analysis. **(D)** Results of p65 DNA methylation statistical analysis. (Model vs Control: *^###^P<*0.001; Positive, TPH vs Model: *P<0.05, **P<0.01; *n*=3).

As shown in **Figure 7A**, a total of 7 CPG sites within the TNF-*α* gene were methylated. The methylation rates were 40.0% in the control group, 17.1% in the model group, 31.4% in the positive group, and 30.0% in the TPH administration group. According to **Figure 7C**, the methylation rates in the positive group and the TPH group were significantly higher than that of the model group (*P<*0.05) and comparable to the control group. At this methylation level, the protein cannot bind to DNA, thereby silencing transcription and preventing tumor gene expression, which contributes to the therapeutic efficacy of the drug.

As shown in **Figure 7B**, a total of 29 CPG sites within the p65 gene were methylated. The methylation rates were 40.9% in the control group, 14.7% in the model group, 31.5% in the positive group, and 27.6% in the TPH administration group. After drug administration, the methylation status of the genes was altered. As shown in **Figure 7D**, the methylation rates in the positive group and the TPH group were significantly higher than that of the model group (*P*<0.05). This hypermethylation of the CPG islands inhibits the expression of tumor-related genes, thereby reducing tumor development.

### 3.6 Qualitative analysis of triterpenoids in TP

**Figure 8:**
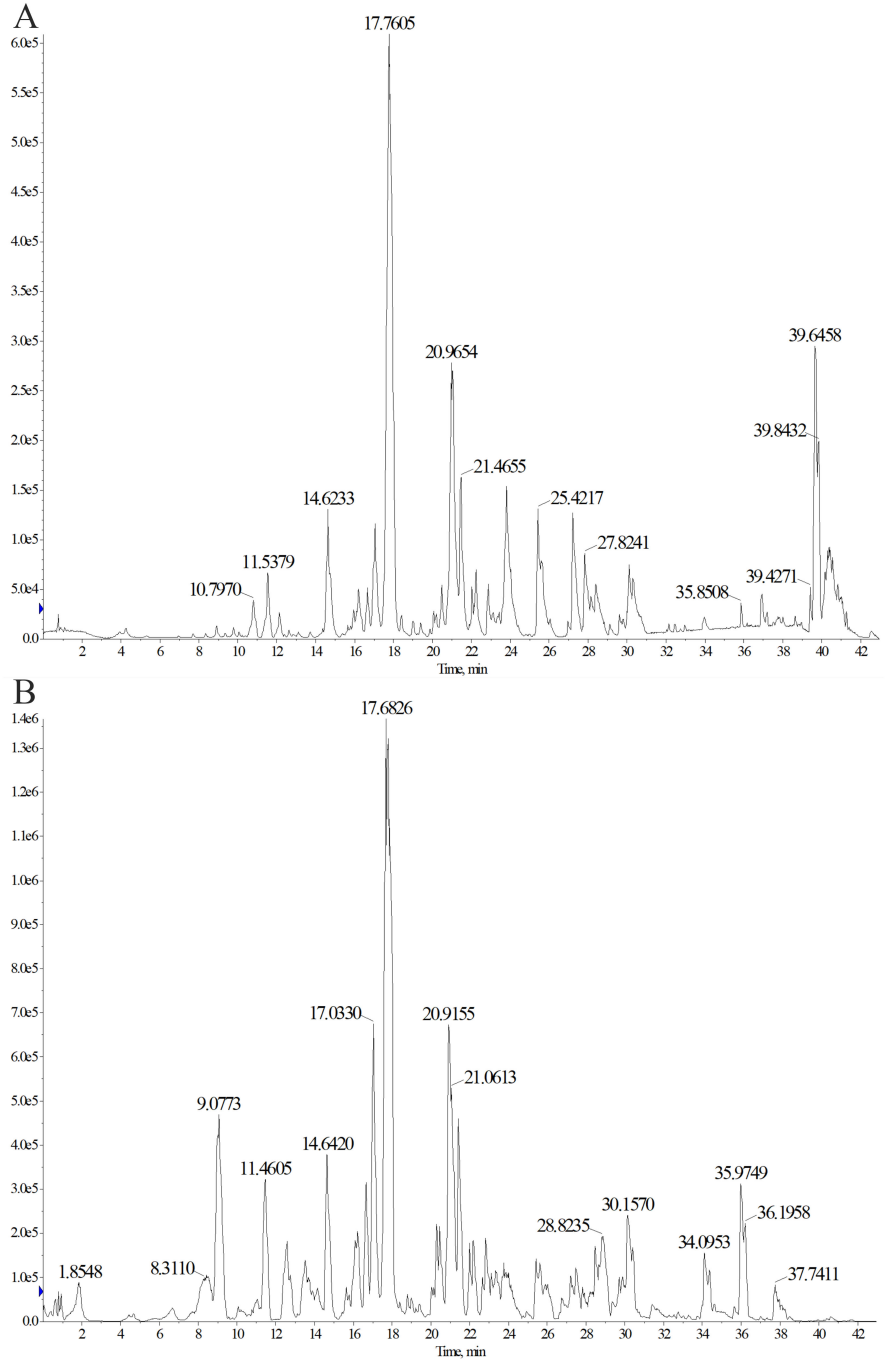
Determination of triterpenoids in TP by ultrasonic-assisted method. Chromatogram (BPC) in positive **(A)** and negative **(B)** ion modes of triterpenoids in TP.

**Figure 9:**
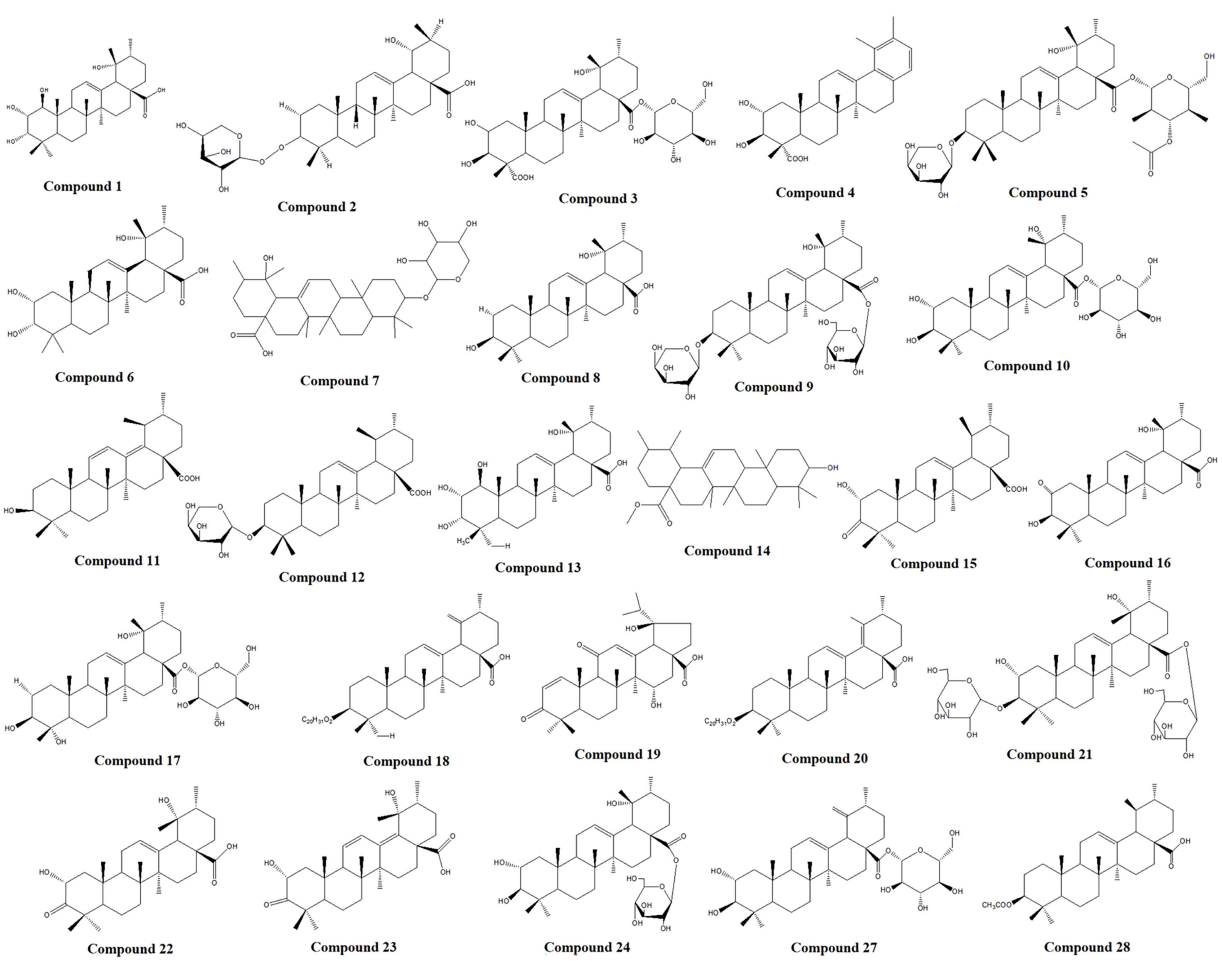
Chemical structure of triterpenoids in TP.

To identify the main triterpenoid components in TP, qualitative analysis was performed using UPLC-Q-TOF-MS. The base peak chromatograms (BPC) in both positive and negative ion modes are shown in **Figure 8**. Through comparison and analysis, a total of 26 triterpenoids were identified (**Table 1**), predominantly pentacyclic triterpenoids. These include 23 ursane-type triterpenoids, 2 oleanane-type triterpenoids, and 1 lupane-type triterpenoid. Most of these compounds have been previously reported in S. L. Notably, compounds 14 and 15 share the same retention time as compounds 25 and 26, respectively, indicating that they are identical compounds. The chemical structures of these compounds are illustrated in **Figure 9**.

**Table 1:** Characterization of triterpenoids compounds from TP by UPLC-Q-TOF-MS.

Compound	t_R_ (min)	Chemical name	Formula	Molecular weight	[M-H]^-^or [M-H]^-^	Major fragment ion	References
1	8.357	1*β*-hydroxyrosic acid	C_30_H_48_O_6_	504.3	505.3	476.3	(4)
2	9.386	3*β*-[(*α*-l-arabinopyranosyl)oxy]-19*α*-hydroxyolean-12-en-28-oic acid	C_33_H_52_O_9_	592.4	593.4	564.4; 484.3	(22)
3	10.808	Sauvissimoside R_1_	C_36_H_56_O_12_	680.4	681.5	340.3	(23)
4	12.172	2*α*,3*β*-dihydroxy-28-norurs-12,17,19 (20),21-tetraen-23-oic acid	C_29_H_40_O_4_	452.6	453.8	153.1	(22)
5	12.654	Sanguisorbins E	C_45_H_72_O_12_	804.5	805.4	689.4; 631.4	–
6	16.207	Euscaphic acid	C_30_H_48_O_5_	488.4	489.4	471.4; 455.4	(4)
7	17.047	Ziyuglycoside II	C_35_H_56_O_8_	604.8	605.4	473.4; 455.4	(4)
8	17.798	3*β*,19*α*- dihydroxyurs-12-en-28-oic acid	C_30_H_48_O_4_	472.4	473.4	456.4; 438.3	–
9	21.008	Ziyuglycoside I	C_41_H_66_O_13_	766.5	765.5	652.4; 437.3	(24)
10	22.872	Rosamutin	C_36_H_58_O_10_	650.4	651.4	471.3; 453.3	(25)
11	23.818	3*β*-hydroxyurs-11,13 (18)-dien-28-oic acid	C_30_H_46_O_3_	454.3	455.3	437.4	(4)
12	27.853	Sanguisorbins B	C_35_H_56_O_7_	588.4	589.4	437.3; 409.3	–
13	28.441	1*β*,2*α*,3*α*,19*α*-tetrahydroxyurs-12-en-28-oic acid	C_31_H_52_O_6_	520.4	521.3	455.4; 437.3	(24)
14^*^	30.124	Ursolic acid methyl ester	C_30_H_46_O_4_	470.3	471.3	435.3; 338.3	(4)
15^*^	30.367	Pomeranic acid	C_30_H_46_O_4_	470.3	471.3	453.3; 301.1	(4)
16	34.033	2-oxopomolic acid	C_30_H_46_O_5_	486.3	487.1	463.1; 439.4	(26)
17	35.862	niga-ichigoside F1	C_35_H_56_O_10_	636.4	637.3	455.4; 376.3	(23)
18	39.422	3*β*-[(*α*-L-arabinopyranosyl)oxy]-urs-12,19(29)-dien -28-oic acid	C_51_H_80_O_4_	757.2	758.2	628.2; 371.1	(22)
19	9.062	lup-12-en-15,19-diol-3,11-dioxo-28-oic acid	C_30_H_42_O_6_	498.3	497.4	487.3	–
20	11.449	3*β*-[(*α*-L-arabinopyranosyl) oxy]urs-12, 18-dien-28-oic acid	C_50_H_76_O_4_	741.2	740.5	723.5	(22)
21	16.217	3-O-*β*-D-glucopyranosyl-2*α*,19*α*-dihydroxyurs-12-en-28-oic acid *β*-D-glucopyranosyl ester	C_42_H_68_O_15_	812.9	811.5	695.4; 577.3	(4)
22	22.847	2*α*,19*α*-dihydroxy-3-oxo-12-ursen-28-oic acid	C_30_H_46_O_5_	486.7	485.3	449.2; 311.2	(4)
23	25.449	2*α*,19*α*-dihydroxy-3-oxo-urs-11,13(18)-dien-28-oic acid	C_30_H_44_O_5_	484.3	483.3	311.2	(4)
24	28.865	2*α*,3*β*,19*α*-trihydr-oxyurs-12-en-28-oic acid *β*-D-galactopyranosyl ester	C_36_H_58_O_10_	650.4	649.4	571.4; 507.3	–
25^*^	30.198	Ursolic acid methyl ester	C_30_H_46_O_4_	470.3	469.3	433.3	(4)
26^*^	30.431	Pomeranic acid	C_30_H_46_O_4_	470.3	469.4	347.2; 293.2	(4)
27	34.134	2*α*,3*α*-dihydroxyurs-12,19(29)-dien-28-oic acid *β*-D-glucopyranosyl ester	C_36_H_56_O_9_	632.4	631.5	501.4; 455.4	–
28	37.763	3-O-acetylursolic acid	C_32_H_50_O_4_	498.7	497.4	255.2	(27)

^*^ is expressed as a repeating compound.

### 3.7 Network pharmacological analysis of TP

**Figure 10:**
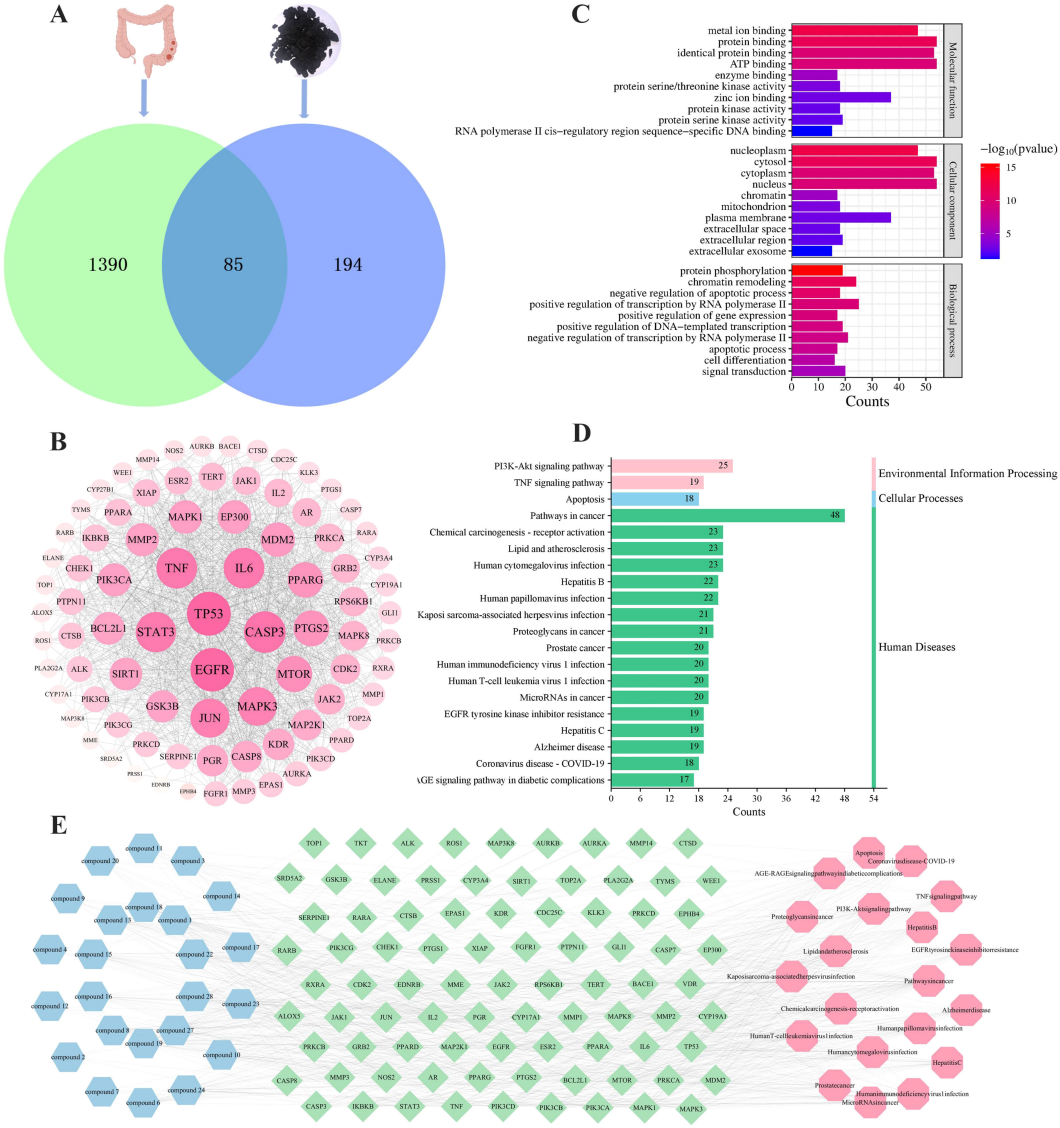
Network pharmacology analysis of TP anti-colon cancer. **(A)** Venn diagram of potential anti-colon cancer targets. **(B)** PPI network of common targets between colon cancer and TP. The darker in color and the larger in circle represented a higher degree. **(C)** Top 10 GO terms of hub genes. **(D)** Top 20 KEGG enrichment results of co-targets. **(E)** “Component-target-pathway” map of TP. Blue represented the TP components, green represented the common targets, and pink represented the pathways.

To elucidate the mechanism of action and identify the potential active compounds of TP, we conducted network pharmacological analysis following the characterization of triterpenoids. According to the SwissTargetPrediction database, 24 of the 26 identified triterpenoids in TP were predicted to have potential therapeutic effects. After removing duplicate targets, a total of 279 target genes were obtained. Additionally, using “colon cancer” as the keyword, we screened 1475 targets from the OMIM and GeneCards databases. By intersecting the drug targets and disease targets, we identified 85 genes as potential cross-targets for TP against colon cancer (**Figure 10A**). **Figure 10B** visually illustrates the PPI network among these targets. The size of each node represents the importance of its core target. The results showed that TP53, EGFR, CASP3, IL-6, TNF, and STAT3 were centrally located in the PPI network, indicating their involvement in the anti-colon cancer process of TP. GO enrichment analysis revealed that TP is associated with molecular functions such as protein binding and ATP binding, as well as biological processes like chromatin remodeling and positive regulation of transcription by RNA polymerase II (**Figure 10C**). KEGG pathway analysis further identified the key pathways through which TP compounds exert their anti-colon cancer effects, with the top 20 pathways including the PI3K-AKT signaling pathway and TNF signaling pathway (**Figure 10D**). Based on these findings, we constructed a “component-target-pathway” network (**Figure 10E**). This network illustrates that the core components of TP (compounds 1, 8, 13, 15, 16, 18, 19, 22, 27, and 28) act against colon cancer by targeting TP53, EGFR, CASP3, IL-6, TNF, STAT3, and key signaling pathways such as PI3K-AKT and TNF signaling. Given the results of network pharmacology and protein-level analysis, TNF-*α* was selected as the focus for further study.

### 3.8 Molecular docking analysis of key active compounds in TP

**Figure 11:**
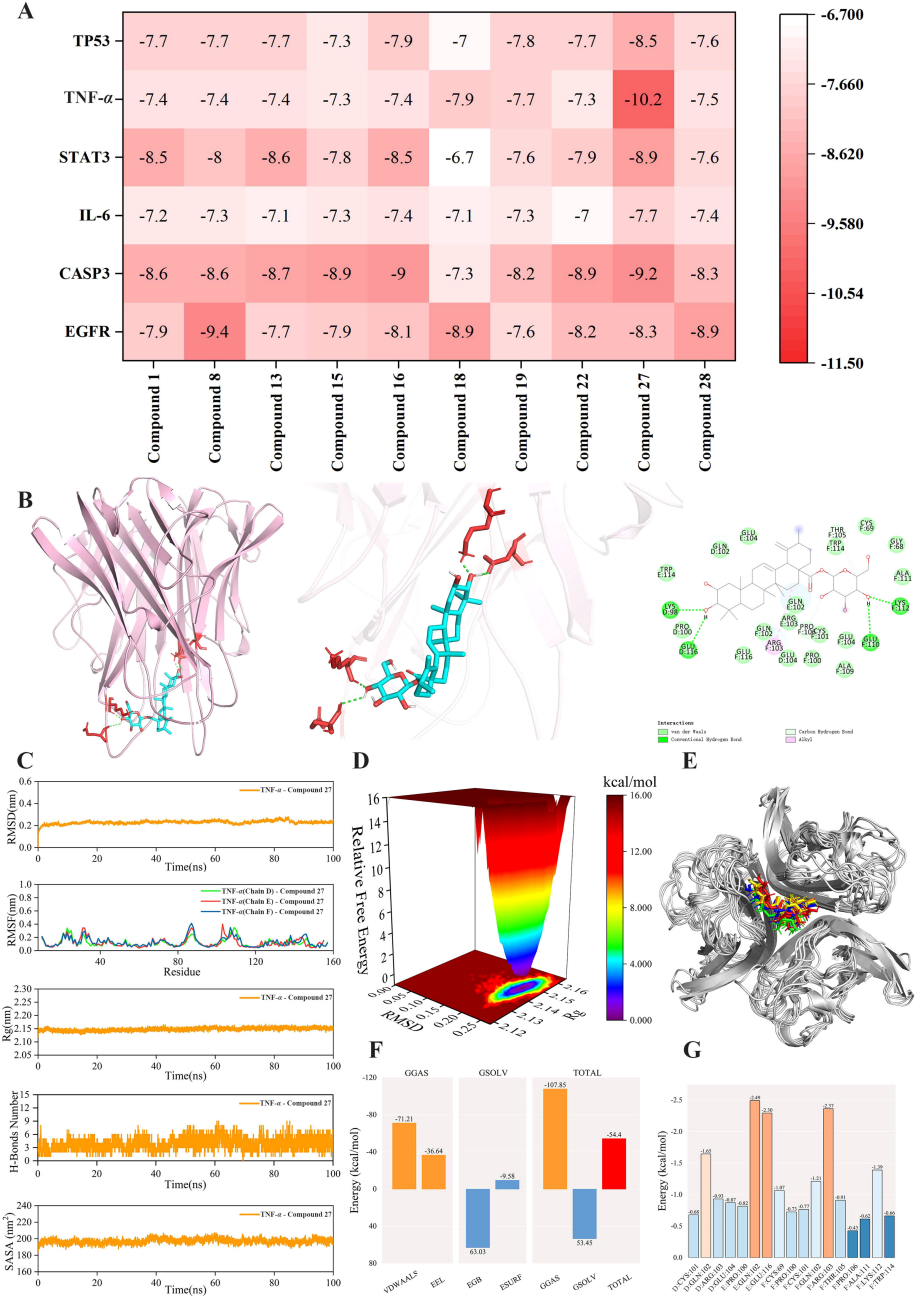
Molecular docking and molecular dynamics simulation analysis of TNF-*α* - Compound 27. **(A)** Docking heat map of 10 main active compounds in TP and 6 core targets. The deeper the red color, the lower the binding energy and the stronger the binding ability. **(B)** Molecular model of TNF-*α* - Compound 27 interaction. **(C)** RMSD curve, RMSF curve, Rg curve, hydrogen bond number fluctuation curve and SASA curve. **(D)** Free energy distribution diagram. **(E)** Comparison of the structure of the complex at five moments of molecular dynamics simulation 0,25,50,75,100ns(In the figure, the small molecules in red, green, blue, yellow and orange correspond to the molecular structure of compound 27 at five times, respectively, 0,25,50,75,100ns). **(F)** Average binding free energy. **(G)** Energy contribution of amino acid residues involved in compound 27 binding in TNF protein.

To identify the main active compounds in TP, molecular docking analysis was performed based on the results of the PPI and “component-target-pathway” network. **Figure 11A** presents the heat map and binding energy values of the compounds. The results indicated that 10 compounds exhibited binding energies lower than -5 kcal/mol, with most having binding energies below -7 kcal/mol. Notably, compound 27 had the highest average binding energy, particularly with TNF-*α*, reaching -10.2 kcal/mol. The interaction forces were further characterized using PyMOL and Discovery Studio, and the molecular model with the lowest binding energy was visualized. As shown in **Figure 11B**, the binding energy between TNF-*α* and compound 27 was -10.2 kcal/mol, indicating a strong docking affinity. The interactions between TNF-*α* and compound 27 were primarily mediated by hydrogen bonds and hydrophobic interactions. Specifically, LYS112 (F chain), GLU110 (F chain), LYS98 (D chain), and GLU116 (D chain) on TNF-*α* formed hydrogen bonds with compound 27. Additionally, PRO106 (F chain) on TNF-*α* formed unconventional hydrogen bonds with compound 27, while ARG103 on the TNF-*α* (F) chain formed a hydrophobic bond with compound 27. In summary, the molecular docking results reveal that compound 27 is a key active component in TP’s prevention of colon cancer, with TNF-*α* likely playing a crucial role in this process.

### 3.9 Molecular dynamics simulations of protein-ligand complexes

To investigate the stability and dynamic interactions of protein-ligand complexes, molecular dynamics simulations were performed to validate the docking results. The stability analysis is shown in **Figure 11C**. The RMSD and RMSF curves of the complex fluctuated within a range of 1 nm, indicating stable conformational dynamics. The Rg curve fluctuated within a range of 2.15 nm and remained stable throughout the simulation. To further explore the hydrogen bond properties of the complex binding sites, the number of main hydrogen bonds stabilizing the ligand-protein interactions was calculated. The number of hydrogen bonds between TNF-*α* and compound 27 stabilized at 3–5 within 0–50 ns and 3–6 within 50–100 ns, with the hydrogen bond curve fluctuating steadily. The SASA curve of the TNF-*α* - compound 27 complex remained stable throughout the simulation, with no significant fluctuations and a variation range of 200 nm². Collectively, these results indicate that the TNF-*α* - compound 27 complex exhibits high stability. **Figure 11D** shows the free energy distribution of the TNF-*α* - compound 27 complex, revealing a single minimum energy cluster with a concentrated energy distribution. This suggests that the complex formed between TNF-*α* and compound 27 is highly stable. The binding stability of the complex was further examined by comparing the conformations at five time points (0, 25, 50, 75, and 100 ns). As shown in **Figure 11E**, TNF-*α* and compound 27 remained bound to the same position at all five time points without significant changes, indicating excellent binding stability. After the complex system reached equilibrium, the average binding free energy of TNF-*α* - compound 27 was calculated using the MM/GBSA method. **Figure 11F** shows that the average binding free energy of TNF-*α* - compound 27 is -54.4 kcal/mol, indicating a strong binding affinity. As depicted in **Figure 11G**, compound 27 formed favorable interactions with specific amino acid residues in the TNF protein, including GLN102 in the E chain (-2.49 kcal/mol), GLU116 in the F chain (-2.30 kcal/mol), and ARG103 in the F chain (-2.37 kcal/mol). These residues play a crucial role in stabilizing the TNF-*α* - compound 27 complex. Overall, the results demonstrate that TNF-*α* and compound 27 exhibit a stable binding interaction.

## 4 Discussion

This experiment successfully established a mouse model of colon cancer, in the process of modeling through the analysis of several indicators of colon cancer, the mice showed weight loss, diarrhea, occult blood in stool, blood in stool, and other symptoms. In addition, the general morphological observation of colonic tissue, intestinal mass index, and HE histopathological changes were used to measure the degree of colonic mucosal injury in mice, to evaluate the therapeutic effect of drugs on colonic tissue injury in mice. Compared with the control group, the colon length of mice in the model group was significantly shortened, the intestinal mass was significantly reduced, and there was obvious tumor formation. The tumor size was also about 4 mm. The size and number of tumors on the colon in the TR group and the TP group were significantly reduced compared with the model group in a dose-dependent manner. HE staining showed that the intestinal mucosal adenoma became smaller and the inflammation was reduced to different degrees. The tumor inhibition effect of the TP group was significantly better than that of the TR group. These results indicate that the triterpenoids before and after processing have a certain preventive effect on colon cancer in mice, but the degree of inhibition is different. Due to the invasiveness of the colon cancer model, some mice died during the experiment, resulting in a small sample size and limitations.

Studies have shown that abnormal proliferation of colon cells is closely related to the occurrence and development of tumors, and tumor proliferation markers can reflect the occurrence and development of tumors (28). Among them, Ki67 exists in every cycle of cells, and can be strongly expressed in tumor cells, but can hardly be detected in normal cells. Its expression level has important prognostic significance in colon cancer, so it can be used to evaluate the survival and progression of cancer (29). In addition, PCNA is a non-histamine nuclear protein whose expression is related to the degree of malignancy, vascular invasion, distal metastasis, and survival rate of tumors, and is a specific marker of cell division (30). PCNA can effectively reflect the proliferation activity of cells, to objectively evaluate the proliferation status of tumor cells. In addition, apoptosis also plays a crucial role in the process of tumorigenesis control and can be used to predict cancer progression. In this study, through the detection of Ki67 and PCNA index and TUNEL apoptosis fluorescence intensity in tumor tissue, it was shown that the expression of Ki67 and PCNA protein in tumor tissue of mice in the model group was high, while the TUNEL apoptosis fluorescence intensity was very low, indicating that tumor cells in tumor tissue had good proliferation ability and little apoptosis. However, TS and Positive drugs can significantly reverse the trend of the above indexes, and the low-dose group and high-dose group have different degrees of reverse regulation ability, which is dose-dependent, indicating that tumor cells in tumor tissue had high proliferation ability and low apoptosis.

The occurrence and progression of colon cancer involve multiple mechanisms. Research shows that S.L. enhances the 5-fluorouracil sensitivity and overcomes chemoresistance in 5-fluorouracil-resistant colorectal cancer cells via Ras/MEK/ERK and PI3K/Akt pathways (31). The Wnt/*β*-catenin signaling pathway also plays an important role in colon cancer. Ursolic acid may inhibit the malignant phenotype, induce apoptosis, and arrest the cell cycle of CRC, by attenuating the Wnt/*β*-catenin signaling axis (32). p53 is an important tumor suppressor gene, and the p53 protein encoded by it plays a key role in cell cycle regulation, DNA repair and apoptosis. Research shows that maslinic acid induces apoptosis in human HT29 colon-cancer cells through the JNK-Bid-mediated mitochondrial apoptotic pathway via the activation of p53 (33). TNF-*α* and NF-κB play crucial roles in the development and progression of colitis as well as the development of colon cancer (34). TNF-*α*, primarily produced by immune cells, activates the NF-κB signaling pathway, which in turn regulates the expression of various inflammation-related genes, thereby enhancing the immune response (35). Activated NF-κB responds to danger signals released by inflammatory cytokines, leading to the production of p65, COX-2, iNOS, and other cytokines. These molecules not only amplify the inflammatory response but also promote cancer cell growth (36). TNF-*α* and NF-κB cooperate in the immune response, jointly promoting inflammation and immune cell activation, thus forming a positive feedback loop (37). As a transcription factor, NF-κB can also regulate the expression of TNF-*α* and other inflammatory factors, further driving the inflammatory response (38). This TNF-*α*/NF-κB-mediated inflammatory response is key to colitis development and strongly associated with colon cancer progression (39). In our study, the model group exhibited increased expression levels of NF-κB and elevated contents of inflammatory cytokines. However, these symptoms were significantly reduced following drug administration. This indicates that the drug intervention inhibits NF-κB signaling pathway activation by downregulating the expression of p65, COX-2, TNF-*α*, and iNOS proteins in colon tissue. This finding suggests that the preventive effect of ST on AOM/DSS-induced colon cancer is primarily mediated through the NF-κB signaling pathway. ST can reduce the protein expression levels of TNF-*α*, NF-κB p65, COX-2, and iNOS in colon tissue in a dose-dependent manner and significantly inhibit the abnormal activation of the NF-κB signaling pathway. Our findings demonstrate that triterpenoids, as biologically active phytochemicals, exert potent chemopreventive effects through multimodal regulation of this pathway. Triterpenoids significantly downregulate TNF-*α* production and inhibit NF-κB activation in a dose-dependent manner, thereby exerting anti-inflammatory effects. Moreover, triterpenoids also suppress the expression of downstream molecules such as COX-2 and the activity of iNOS.

To further clarify the transcriptional regulation mechanism of the TNF and NF-κB signaling pathway in colon cancer, more in-depth studies have been carried out at the gene level. Western-blot experiments has proved that the effect of the TPH administration group is better, so the TPH group is selected as the administration group for subsequent experiments. In addition, we selected the tumor necrosis factor TNF-*α* and nuclear translocation factor NF-κB p65 as the main DNA methylation indicators. Changes in the level and pattern of DNA methylation are an important factor in tumorigenesis (40). Abnormal changes in DNA methylation status are common in various tumors, and abnormal DNA methylation status is one of the important characteristics of tumors. Therefore, DNA methylation is of great significance in the early diagnosis and prognosis evaluation of tumors (41). In this study, mouse colon tissue was purified using BSP, and positive plasmids were obtained for subsequent sequencing analysis. Comparison of the methylation rates across different groups revealed that tumor cells are typically activated in a hypomethylated state, which is associated with pro-tumorigenic activity. Conversely, administration of TPH induced a hypermethylated state, which was associated with the inhibition of tumor cell activation. This finding indicates that TP can suppress tumor genes at the genetic level, thereby inhibiting tumor initiation and progression. The results of DNA methylation analysis further demonstrate that triterpenoids exert epigenetic regulatory effects by modulating DNA methylation status. In the model group, a hypomethylation state was observed, which is typically associated with pro-tumorigenesis. In contrast, TPH treatment significantly increased the methylation level of the TNF-*α*/NF-κB binding site, inducing a hypermethylated state. This hypermethylation is closely correlated with the transcriptional silencing of oncogenic pathways. These findings suggest that TPH inhibits the expression of inflammatory and tumor-related genes by inducing hypermethylation at key regulatory sites, thereby exerting its anti-tumor effects.

The triterpenoids components were qualitatively analyzed by UPLC-Q-TOF-MS. High-resolution mass accuracy (<5ppm) and dual-ion mode detection enhance the coverage of triterpenoids, especially for those with lower ionization efficiency in a single mode. We employed UPLC-Q-TOF-MS to identify 26 triterpenoids in TP. Subsequently, active compounds were screened using network pharmacology. Molecular docking technology, a computational chemistry method that simulates the binding process between ligands and receptors, was utilized to predict their binding modes and affinities (42). Generally, a docking score <0 kcal/mol indicates spontaneous binding between the compound and target, a score <-5 kcal/mol suggests good binding affinity, and a score <-7 kcal/mol is considered to indicate strong binding affinity (43). Our molecular docking results revealed that compound 27 exhibited high binding energy with TNF-*α*. Further molecular dynamics simulations demonstrated that the TNF-*α* - compound 27 complex was stable. Collectively, these findings suggest that compound 27 in TP may contribute to the prevention of colon cancer by forming a stable complex with TNF-*α*, thereby downregulating TNF-*α* and inhibiting the NF-κB signaling pathway. The calculation and prediction results need to be further confirmed in combination with *in vitro* activity verification.

## 5 Conclusions

In this study, TR and TP have different degrees of prevention and treatment effects on AOM/DSS-induced colon cancer mice. They might reduce the proliferation of colon cancer cells by binding to TNF-*α*, down-regulating TNF-*α*, inhibiting the NF-κB signaling pathway, suppressing the CPG island from being in a hypermethylated state, and affecting the binding of proteins to the DNA promoter region. In conclusion, this study provides preliminary methodological and theoretical foundations for the search of new anti-tumor drugs, and also provides a new idea for the development and utilization of S.L. and its processed products.

## Data Availability

The datasets presented in this study can be found in online repositories. The names of the repository/repositories and accession number(s) can be found in the article/supplementary material.

## References

[1] Cai Z Li W Wang H Yan W Zhou Y Wang G. Anti-tumor and immunomodulating activities of a polysaccharide from the root of *Sanguisorba officinalis* L. Int J Biol Macromolecules. (2012) 51:484–8. doi: 10.1016/j.ijbiomac.2012.05.029 22659266

[2] Li W Yang CJ Wang LQ Wu J Dai C Yuan YM. A tannin compound from *Sanguisorba officinalis* blocks Wnt/*β*-catenin signaling pathway and induces apoptosis of colorectal cancer cells. Chin Med. (2019) 14:22. doi: 10.1186/s13020-019-0244-y 31164916 PMC6544925

[3] Lkhagvasuren K Kim JK. Ziyuglycoside II induces caspases-dependent and caspases-independent apoptosis in human colon cancer cells. Toxicol In Vitro. (2019) 59:255–62. doi: 10.1016/j.tiv.2019.04.028 31029785

[4] Wang Z Yang CJ Wu L Sun JH Wang Z Wang Z. Variation of Saponins in *Sanguisorba officinalis* L. before and after Processing (Paozhi) and Its Effects on Colon Cancer Cells *In Vitro* . Molecules. (2022) 27:9046. doi: 10.3390/molecules27249046 36558181 PMC9785891

[5] Su XD Guo RH Li HX Ma JY Kim YR Kim YH. Anti-allergic inflammatory components from *Sanguisorba officinalis* L. Bioorg Med Chem Lett. (2018) 28:2210–6. doi: 10.1016/j.bmcl.2018.04.033 29759725

[6] Shah SC Itzkowitz SH. Colorectal cancer in inflammatory bowel disease: mechanisms and management. Gastroenterology. (2022) 162:715–730.e3. doi: 10.1053/j.gastro.2021.10.035 34757143 PMC9003896

[7] Koklesova L Liskova A Samec M Qaradakhi T Zulli A Smejkal K. Genoprotective activities of plant natural substances in cancer and chemopreventive strategies in the context of 3P medicine. EPMA J. (2020) 11:261–87. doi: 10.1007/s13167-020-00210-5 PMC7272522 32547652

[8] Macharia JM Mwangi RW Rozmann N Zsolt K Varjas T Uchechukwu PO. Medicinal plants with anti-colorectal cancer bioactive compounds: Potential game-changers in colorectal cancer management. Biomedecine pharmacotherapie. (2022) 153:113383. doi: 10.1016/j.biopha.2022.113383 35820316

[9] Huang Y Lai Y Chen L Fu K Shi D Ma X. Danshensu enhances autophagy and reduces inflammation by downregulating TNF-*α* to inhibit the NF-κB signaling pathway in ischemic flaps. Phytomedicine. (2025) 137:156378. doi: 10.1016/j.phymed.2025.156378 39818119

[10] Ramakrishnan SK Zhang H Ma X Jung I Schwartz AJ Triner D. Intestinal non-canonical NFκB signaling shapes the local and systemic immune response. Nat Commun. (2019) 10:660. doi: 10.1038/s41467-019-08581-8 30737385 PMC6368617

[11] Dzhalilova D Zolotova N Fokichev N Makarova O. Murine models of colorectal cancer: the azoxymethane (AOM)/dextran sulfate sodium (DSS) model of colitis-associated cancer. PeerJ. (2023) 11:e16159. doi: 10.7717/peerj.16159 37927787 PMC10624171

[12] Zhong Y Li XY Zhou F Cai YJ Sun R Liu RP. Ziyuglycoside II inhibits the growth of digestive system cancer cells through multiple mechanisms. Chin J Nat Med. (2021) 19:351–63. doi: 10.1016/S1875-5364(21)60033-X 33941340

[13] Wang L Chen Q Song H Xing W Shi J Li Y. The anti-colorectal cancer effect and metabolites of Agrimonia pilosa Ledeb. J ethnopharmacology. (2024) 329:118146. doi: 10.1016/j.jep.2024.118146 38604512

[14] Fang H Xie X Liu P Rao Y Cui Y Yang S. Ziyuglycoside II alleviates cyclophosphamide-induced leukopenia in mice via regulation of HSPC proliferation and differentiation. BioMed Pharmacother. (2020) 132:110862. doi: 10.1016/j.biopha.2020.110862 33069969

[15] Liu MP Liao M Dai C Chen JF Yang CJ Liu M. *Sanguisorba officinalis* L synergistically enhanced 5-fluorouracil cytotoxicity in colorectal cancer cells by promoting a reactive oxygen species-mediated, mitochondria-caspase-dependent apoptotic pathway. Sci Rep. (2016) 6:34245. doi: 10.1038/srep34245 27671231 PMC5037464

[16] Zhang W Peng C Yan J Chen P Jiang C Sang S. *Sanguisorba officinalis* L. suppresses 5-fluorouracil-sensitive and-resistant colorectal cancer growth and metastasis via inhibition of the Wnt/*β*-catenin pathway. Phytomedicine. (2022) 94:153844. doi: 10.1016/j.phymed.2021.153844 34785413

[17] Zhang W Peng C Shen X Yuan Y Zhang W Yang C. A bioactive compound from *sanguisorba officinalis* L. Inhibits cell proliferation and induces cell death in 5-fluorouracil-sensitive/resistant colorectal cancer cells. Molecules. (2021) 26:3843. doi: 10.3390/molecules26133843 34202548 PMC8270258

[18] Zhang L Li J Huo Y Yang W Chen J Gao Z. Ultrasonic extraction and antioxidant evaluation of oat saponins. Ultrasonics sonochemistry. (2024) 109:106989. doi: 10.1016/j.ultsonch.2024.106989 39059252 PMC11327440

[19] Liu YJ Tang B Wang FC Tang L Lei YY Luo Y. Parthenolide ameliorates colon inflammation through regulating Treg/Th17 balance in a gut microbiota-dependent manner. Theranostics. (2020) 10:5225–41. doi: 10.7150/thno.43716 PMC7196297 32373209

[20] Wu X Xu L Li X Zhou Y Han X Zhang W. A HER2-targeting antibody-MMAE conjugate RC48 sensitizes immunotherapy in HER2-positive colon cancer by triggering the cGAS-STING pathway. Cell Death disease. (2023) 14:550. doi: 10.1038/s41419-023-06073-8 37620320 PMC10449775

[21] Wu X Zheng Z Guo T Wang K Zhang Y. Molecular dynamics simulation of lentinan and its interaction with the innate receptor dectin-1. Int J Biol macromolecules. (2021) 171:527–38. doi: 10.1016/j.ijbiomac.2021.01.032 33428957

[22] Liu X Cui Y Yu Q Yu B. Triterpenoids from sanguisorba officinalis. Phytochemistry. (2005) 66:1671–9. doi: 10.1016/j.phytochem.2005.05.011 15979111

[23] Xia H Zhang XL Wang GH Tong YC He L Wang HF. Chemical constituents from Barringtonia racemosa. Zhongguo Zhong yao za zhi. (2016) 41:2460–5. doi: 10.4268/cjcmm20161315 28905569

[24] Wei FS Yang CJ Wu L Sun JH Wang ZY Wang ZB. Simultaneous determination and pharmacokinetics study of three triterpenes from *sanguisorba officinalis* L. @ in rats by UHPLC-MS/MS. Molecules (Basel Switzerland). (2022) 27:5412. doi: 10.3390/molecules27175412 36080179 PMC9458004

[25] Adnyana IK Tezuka Y Awale S Banskota AH Tran KQ Kadota S. six new triterpene glucosides from the seeds of Combretum quadrangulare. Chem Pharm bulletin. (2000) 48:1114–20. doi: 10.1248/cpb.48.1114 10959573

[26] Zhang XY Li W Wang J Li N Cheng MS Koike K. Protein tyrosine phosphatase 1B inhibitory activities of ursane-type triterpenes from Chinese raspberry, fruits of Rubus chingii. Chin J Natural Medicines. (2019) 17:15–21. doi: 10.1016/S1875-5364(19)30004-4 30704618

[27] Jo Y Suh J Shin MH Jung JH Im KS. Jacaranone and related compounds from the fresh fruits of Ternstroemia japonica and their antioxidative activity. Arch Pharm Res. (2005) 28:885–8. doi: 10.1007/BF02973871 16178411

[28] Dekker E Tanis PJ Vleugels JLA Kasi PM Wallace MB. Colorectal cancer. Lancet. (2019) 394:1467–80. doi: 10.1016/S0140-6736(19)32319-0 31631858

[29] Aladhraei M Kassem Al-Thobhani A Poungvarin N Suwannalert P. Association of XPO1 overexpression with NF-κB and ki67 in colorectal cancer. Asian Pac J Cancer Prev. (2019) 20:3747–54. doi: 10.31557/APJCP.2019.20.12.3747 PMC7173379 31870117

[30] Tan G Huang C Chen J Zhi F. HMGB1 released from GSDME-mediated pyroptotic epithelial cells participates in the tumorigenesis of colitis-associated colorectal cancer through the ERK1/2 pathway. J Hematol Oncol. (2020) 13:149. doi: 10.1186/s13045-020-00985-0 33160389 PMC7648939

[31] Zhao H Tang S Tao Q Ming T Lei J Liang Y. Ursolic acid suppresses colorectal cancer by down-regulation of Wnt/*β*-Catenin signaling pathway Activity. J Agric Food Chem. (2023) 71:3981–93. doi: 10.1021/acs.jafc.2c06775 36826439

[32] Zhang W Ou L Peng C Sang S Feng Z Zou Y. *Sanguisorba officinalis* L. enhances the 5-fluorouracil sensitivity and overcomes chemoresistance in 5-fluorouracil-resistant colorectal cancer cells via Ras/MEK/ERK and PI3K/Akt pathways. Heliyon. (2023) 9:e16798. doi: 10.1016/j.heliyon.2023.e16798 37484409 PMC10360953

[33] Reyes-Zurita FJ Pachón-Peña G Lizárraga D Rufino-Palomares EE Cascante M Lupiáñez JA. The natural triterpene maslinic acid induces apoptosis in HT29 colon cancer cells by a JNK-p53-dependent mechanism. BMC Cancer. (2011) 11:154. doi: 10.1186/1471-2407-11-154 21524306 PMC3103477

[34] Luan Y Li Y Zhu L Zheng S Mao D Chen Z. Codonopis bulleynana Forest ex Diels inhibits autophagy and induces apoptosis of colon cancer cells by activating the NF-κB signaling pathway. Int J Mol Med. (2018) 41:1305–14. doi: 10.3892/ijmm.2017.3337 PMC5819931 29286074

[35] Saxena SK Kumar S Maurya VK Nayak D Kaushik S Manchanda RK. Antiviral and anti-inflammatory activity of novel belladonna formulation against Japanese encephalitis virus via inhibition of p65 nuclear translocation and TNF-*α* mediated NF-κB signaling. Biotechnol Genet Eng Rev. (2023) 39:937–59. doi: 10.1080/02648725.2023.2166258 36718919

[36] Zhang J Zhang P Li S Yu T Lai X He Y. Study on the effect and mechanism of Lacticaseibacillus rhamnosus AFY06 on inflammation-associated colorectal cancer induced by AOM/DSS in mice. Front Microbiol. (2024) 15:1382781. doi: 10.3389/fmicb.2024.1382781 38572238 PMC10987852

[37] Hayden MS Ghosh S. Regulation of NF-κB by TNF family cytokines. Semin Immunol. (2014) 26:253–66. doi: 10.1016/j.smim.2014.05.004 PMC4156877 24958609

[38] Yin X Gao Q Li C Yang Q Dong HL Li Z. Leonurine alleviates vancomycin nephrotoxicity via activating PPARγ and inhibiting the TLR4/NF-κB/TNF-*α* pathway. Int immunopharmacology. (2024) 131:111898. doi: 10.1016/j.intimp.2024.111898 38513573

[39] Liu BX Xie Y Zhang J Zeng S Li J Tao Q. SERPINB5 promotes colorectal cancer invasion and migration by promoting EMT and angiogenesis via the TNF-*α*/NF-κB pathway. Int immunopharmacology. (2024) 131:111759. doi: 10.1016/j.intimp.2024.111759 38460302

[40] Jung G Hernández-Illán E Moreira L Balaguer F Goel A. Epigenetics of colorectal cancer: biomarker and therapeutic potential. Nat Rev Gastroenterol hepatology. (2020) 17:111–30. doi: 10.1038/s41575-019-0230-y PMC7228650 31900466

[41] Papanicolau-Sengos A Aldape K. DNA methylation profiling: an emerging paradigm for cancer diagnosis. Annu Rev pathology. (2022) 17:295–321. doi: 10.1146/annurev-pathol-042220-022304 34736341

[42] Huang S. Efficient analysis of toxicity and mechanisms of environmental pollutants with network toxicology and molecular docking strategy: Acetyl tributyl citrate as an example. Sci total environment. (2023) 905:167904. doi: 10.1016/j.scitotenv.2023.167904 37858827

[43] Shang L Wang Y Li J Zhou F Xiao K Liu Y. Mechanism of Sijunzi Decoction in the treatment of colorectal cancer based on network pharmacology and experimental validation. J ethnopharmacology. (2023) 302:115876. doi: 10.1016/j.jep.2022.115876 36343798

